# Minimising barriers to dental care in older people

**DOI:** 10.1186/1472-6831-8-7

**Published:** 2008-03-26

**Authors:** Elena Borreani, Desmond Wright, Sasha Scambler, Jennifer E Gallagher

**Affiliations:** 1 King's College London Dental Institute at Guy's, King's College and St Thomas' Hospitals, Department of Oral Health Services Research & Dental Public Health, Oral Health Workforce & Education Research Group, London, UK; 2 Consultant in Dental Public Health, Lewisham Primary Care Trust, London, UK; 3 Consultant in Dental Public Health, Lambeth Primary Care Trust, London, UK

## Abstract

**Background:**

Older people are increasingly retaining their natural teeth but at higher risk of oral disease with resultant impact on their quality of life. Socially deprived people are more at risk of oral disease and yet less likely to take up care. Health organisations in England and Wales are exploring new ways to commission and provide dental care services in general and for vulnerable groups in particular. This study was undertaken to investigate barriers to dental care perceived by older people in socially deprived inner city area where uptake of care was low and identify methods for minimising barriers in older people in support of oral health.

**Methods:**

A qualitative dual-methodological approach, utilising both focus groups and individual interviews, was used in this research. Participants, older people and carers of older people, were recruited using purposive sampling through day centres and community groups in the inner city boroughs of Lambeth, Southwark and Lewisham in South London. A topic guide was utilised to guide qualitative data collection. Informants' views were recorded on tape and in field notes. The data were transcribed and analysed using Framework Methodology.

**Results:**

Thirty-nine older people and/or their carers participated in focus groups. Active barriers to dental care in older people fell into five main categories: cost, fear, availability, accessibility and characteristics of the dentist. Lack of perception of a need for dental care was a common 'passive barrier' amongst denture wearers in particular. The cost of dental treatment, fear of care and perceived availability of dental services emerged to influence significantly dental attendance. Minimising barriers involves three levels of action to be taken: individual actions (such as persistence in finding available care following identification of need), system changes (including reducing costs, improving information, ensuring appropriate timing and location of care, and good patient management) and societal issues (such as reducing isolation and loneliness). Older people appeared to place greater significance on system and societal change than personal action.

**Conclusion:**

Older people living within the community in an inner city area where NHS dental care is available face barriers to dental care. Improving access to care involves actions at individual, societal and system level. The latter includes appropriate management of older people by clinicians, policy change to address NHS charges; consideration of when, where and how dental care is provided; and clear information for older people and their carers on available local dental services, dental charges and care pathways.

## Background

### Demographic trends and oral health needs

Given the expectation of continuing global population ageing [1], increasing oral and dental health needs of older people [2-5], the challenge of 'maintaining maximum individual and population health' [1,6], and the recognition that 'oral health is a critical component of holistic health' [1], the provision of dental care for older people is vitally important [7]. The population of older people in England is increasing both in absolute numbers and relative to the rest of the population [8-10]. It is recognised that 'demographic, social, economic and political factors' influence the demand for, and supply of, care services [11]. London has a lower proportion of older people compared with the national picture; however, a higher proportion of older people in London 'live in poverty' and 'in poor health', 'experience inadequate housing' and have 'little or no support from family and friends' [12]. Although defined, in this study, as adults aged 65 years and over, older people are not a homogeneous group; and may helpfully be considered in three age-bands, young old (65–74), older old (75–84), and oldest (85 years and over), albeit that their physical and chronological age may not be the same [8,9]. The majority of older people (95%) live in the community with only about 5% living in care homes [8].

### Uptake of dental care

Uptake of dental care amongst older people is poor, both locally and nationally [13]. National Health Service [NHS] data from March 2006 show that the uptake of NHS dental care is highest amongst middle-aged adults and declines with increasing age, with estimated registration rates falling from 56% in the 45–54 year age-band down to 29% amongst adults 75 years and over, in Lambeth [14]. Similar figures are reported for Southwark (54% down to 30%) and Lewisham (58% down to 35%) [14]. Detailed information on uptake of care by age-band is not available as yet under the new dental contract implemented in April 2006 [15]; however, recent evidence suggests that the proportion of adults in England accessing NHS dental care within the past two years has changed little, and if anything is falling [16]. Barriers to dental care amongst adults in general have been well recognized as a result of the seminal work of Finch et al. [17], during the 1980's and subsequent national surveys [2,18]. Dental attendance amongst older people is strongly associated with having some natural teeth, higher social class, and inversely associated with fear [2]. There has been some research into the factors which impact on uptake of dental care amongst older people in the USA [19,20], and within Europe [21].

### Rationale for research and objectives

Given an ageing population, their changing oral health needs and demands and the low uptake of care, it is important to understand older peoples' perception of oral health, what they perceive are the barriers to care and how they feel they may be minimised. This will enable planners, commissioners and providers to provide patient-centred care for older people living in the community. Two of the four main objectives of the study are presented in this paper; first, to identify barriers to dental care perceived by older people in three socially deprived inner-city boroughs; and second, to identify a range of methods for minimising barriers in older people in support of oral health. Their perspectives on oral health and their use of dental healthcare will be reported separately.

## Methods

In line with the objectives of the research, participants recruited to the study were individuals aged 65 years and over, resident in inner city boroughs of Lambeth, Southwark and Lewisham in South London. The older people were living in the community, with or without carers. King's College London Ethics Committee approval was obtained for this study (04/05–129). Participants were recruited through local day centres and community groups for older people and through advertisements in the newsletters of carers associations locally.

Purposive sampling was used to reflect the diversity of the geographical areas, in line with four main criteria: age band, gender, ethnicity and location (borough of residence). This study was carried out utilising a qualitative dual-methodological approach, combining focus groups and individual interviews, to allow for both depth understanding of individual and group experiences and theory generation. Each focus group had a maximum number of six participants and lasted for around an hour, while each individual interview lasted approximately thirty minutes. Informed consent was obtained prior to participation in the research; volunteers were given details about the study, confidentiality and the right to withdraw at any time without any detriment to current or future care. A topic guide was utilised to ensure that key areas were covered in each focus group and interview. All sessions were recorded and transcribed to facilitate the analysis.

In order to facilitate the conversation and explore issues from the participants' point of view, each interview commenced with an open question, where older people were asked to evaluate their own oral health. Later focus groups and individual interviews were also used to test emerging concepts and ideas.

Data analysis was conducted using Framework Methodology [22,23], which is a matrix-based approach to qualitative data management and analysis, successfully and widely used in health services research. It is a systematic approach that treats cases consistently and allows comparisons within and between cases. Data analysis involved three different stages and processes within an analytical hierarchy [24].

First, 'data management', whereby the 'raw data are reviewed, labelled, sorted and synthesised'; second, developing 'descriptive accounts' in which the ordered data are used to 'identify key dimensions, map the range and diversity of each phenomenon and develop classifications and typologies'; third, creating 'explanatory accounts' or 'explanations of why the data take the forms that are found and presented' [24]. Throughout the process the data were checked and rechecked across the levels of abstraction in an iterative process in line with the methodology.

## Results

### Response

Ninety day centres and community groups were contacted by phone and/or e-mail with an invitation to participate in the study. Eleven day centres were visited at which centre managers facilitated recruitment to this study onsite, enabling ten focus groups to be run over a five-month period. Interviews were conducted with carers of older people and informants who preferred this approach. The majority of participants, or older people to whom the findings related were in the 75–84 year age-band (n = 19), followed by 65–74 (n = 15) and 85 year and over (n = 2) (Table 1). People who declined to participate in the study commonly perceived they did not have any dental need because they had complete dentures.

**Table 1:** Demography of respondents participating in qualitative research on minimising barriers to dental care by age, sex, ethnic group and borough of residence

	**Sex**					
**Age groups**	**Male**	**Female**	**Male**	**Female**	**Male**	**Female**

	**Borough of residence**					

	**Southwark**		**Lambeth**		**Lewisham**	

(1) 65–74	-	4	1	2	-	8
(2) 75–84	2	5	2	2	2	6
(3) 85+	2	-	-	-	-	-

	**Ethnic groups**					

White	4	9	3	3+1 carer	2	10
Mixed	-	-	-	-	-	-
Asian	-	-	1 carer	-	-	-
Black	-	-	-	1	-	2
Chinese and other groups	-	-	-	-	-	2+1 carer

Total participants: 13 + 9 + 17 = 39

3 carers:

A1 White British, female, Lambeth

B2 Asian, female, Lewisham

C1 Mixed, male, Lambeth

### Main barriers to dental care

The results of this study suggest that there are five key areas which act as barriers to older people utilising dental care, when a need to do so was perceived. These are outlined in the first column of Table 2. One further issue identified was a lack of perception of need, particularly amongst people with complete dentures. Each of the barriers was identified as a factor which would cause participants to either forgo dental treatment altogether or delay it until they felt it was absolutely necessary (symptomatic attendance). Starting with 'cost' and taking each of the barriers in turn we examine the implications of the barriers themselves and suggestions made for removing these barriers by older people. The issues are illustrated by quotations and related to the literature and the wider context.

**Table 2:** System changes to minimise barriers to dental care in older people in inner-city area characterised by social deprivation

	**Organisational action- to minimise dental care**		
	
**Barriers to Dental Care**	**Dental teams**	**Primary Care Trusts**	**Department of Health**
**Cost of Dental Treatment**	Provide clear information on dental charges to prospective and current patients Ensure patients receive costed treatment options, sensitive discussions in relation to dental treatment and charges and the opportunity to agree course of treatment appropriate to circumstances. this includes the time period over when the care is provided.	Reduction on cost of treatment for older people Free dental check-ups for older people Provide specific information for older people on dental charges Commission free oral screening at day centres	Provide greater financial support for dental treatment in older people (free dental check-ups and/or free dental care or subsidised care) Ensure clear public information on costs and exemptions

**Fear of Dental Treatment**	Take time to explain the treatment and put the patient at ease Keep the surgery environment friendly and welcoming Address stressors such as the noise of dental drills through provision of ear plugs or silent drills Positive image of dentistry	Request that postgraduate deaneries consider 'care for the older person' as an important area for continuing professional education	Support training on caring for oral health in older people, including the management of dental anxiety and phobia

**Accessibility of Dental Services**	Facilitate the timing of the appointments to minimise indirect costs Ensure older people have appropriate physical access to dental care (e.g. disabled access) Employ dentists to travel around community centres providing outreach services	Proactively commissioning of dental services for older people, both routine and domiciliary care based on need Ensuring care pathways to dental care in general, including domiciliary services	Monitor uptake of NHS dental care by older people and ensure that access to NHS is appropriate

**Availability of Dental Services**	Provide information about the practice and make it available at key locations in the local community – i.e. GP Surgeries, libraries, etc	Commission sufficient care for older people Provide information for older people on local dental services Provide information in GP surgeries and Health Centres on dental services serving older people	Ensure availability of appropriate care Raising awareness of older people's oral health needs across health and social care.

**Characteristics of the Dentist**	Recognise that polite and friendly approach is valued by older people Minimise waiting times Recognise and manage fears relating to past dental care Taking time to talk to patients Provide an atmosphere of unhurried treatments Continue to Provide NHS dental care for older people	Request that postgraduate deaneries consider 'care for the older person' as an important area for continuing professional development	Developing the concept of dental practitioners with a special interest in providing care for older people

### Cost of dental care

The informants in this study included older people who paid NHS charges for dental treatment, those who were partially or fully exempt from NHS charges and people who paid for private treatment.

#### Direct Costs

The actual costs charged to patients through either NHS or private practices for routine check-ups and dental treatments were widely held to be excessive amongst this sample of older people. This was identified as a major cause of infrequent or non-attendance. The majority of participants in this study use NHS dental services. This was largely due to the common perception that private charges were prohibitive. Even those who used the NHS system felt that, unless you were exempt charges, costs could be a barrier to older people receiving pensions.

*If you've got fillings and x-rays you are paying quite a bit of money and as I said when you are a pensioner...it's not that I haven't got anything ...I get two pensions...so therefore I don't get anything – I don't get any benefits or pension credit because I've got two pensions. *(53:90)

In some cases older people did not want to return to a dentist in order to avoid the embarrassment of a situation where they could not afford treatment as demonstrated below:

*My daughter said, 'You should have said that you can't afford it.'; but I didn't like to, so I think I'm not going back. *(41:204)

Amongst those using private dental services there was also the recognition that cost could be a barrier to private patients seeking non-symptomatic treatments:

*I go to private dentists and I just can't afford to go for six-month check-up, it's too expensive. *(36:31).

Dentures were raised as a particular issue for older people. From a cost perspective, some participants considered themselves fortunate to wear dentures, instead of having natural teeth, because of the widely held perception that they needed less dental treatment:

*I'm one of the 'fortunate', if you can call it that way, having a plate on top and bottom, like I said the only times I go is if repairs are needed. *(66:359)

For others, dentures did represent a cost issue for some participants and their peer group. Some indicated that dentures were not worn by their peers because they did not fit properly and alterations or new dentures were too expensive.

*One of the reasons you see a lot of people who don't have their false teeth in is because of finance. *(21:96; 101)

*Don't forget we are all on small pensions and even if you've got a company pension it's still not enough to go and pay £50 or a £150 for a bottom plate. *(21:30)

#### Indirect costs

Indirect costs include expenses other than the dental treatment itself that add to the cost of going to the dentist. The biggest indirect cost identified by this age group was transport. This was a particular issue for people without friends or family to accompany or drive them to appointments. Even where cars were available, it was suggested that public transport may be the cheaper option when additional expenses, such as the London congestion charge (daily charge for driving in central London during the working day) and the cost of parking, were taken into account.

*The one I go to now is the congestion charge; you can't go by car because you can't pay eight pounds every time. I've got to get a bus to go down there. *(23:309)

One of the biggest issues that emerged relating to transport, was the time of the appointment. In London, people over the age of sixty or registered disabled are entitled to a 'freedom pass', which allows them to travel free on London's public transport network after 9 am. When an appointment is too early in the morning, older people are not able to use their free bus pass. Therefore using public transport might represent an additional cost to dental care.

*I got the other week an appointment at quarter past nine, it cost me three pounds to get there because for my bus pass was too early. *(24:332)

#### Fear of the cost of dental treatment

In addition to the direct or indirect cost of treatment which caused a barrier to attendance, fear of the potential cost and the embarrassment of being unable to pay was a barrier to accessing care.

*I'm just afraid of what they are gonna charge. *(12:1012; 1022).

Whilst a small number of participants stated that cost was not an issue in utilising care, the majority of participants stated that cost or the fear of cost was the most significant barrier to utilisation.

### Minimising Barriers Associated with Cost

A range of suggestions was made about how barriers related to the cost of dental treatment could be removed (Table 2). These related to removing or mitigating the direct costs of dental treatment for older people, and particularly of denture related costs; providing more information for older people on the costs of treatment so that decisions can be made according to actual costs rather than through fear of potential costs; and providing more NHS dentists so that all older people have access to NHS dental care should they want or need it. One participant suggested that all NHS dentists should be contracted to see a certain number of older people as a proportion of their patient list.

#### Actual cost

Suggestions relating to the direct costs were primarily focused on decreasing the cost of check-ups and of specific treatments. The vast majority of participants suggested that free dental check-ups should be made available for older patients. This was suggested as a self-evident solution requiring no further explanation.

*Check-ups should be free. *(66:465)

A further suggestion was that if check-ups were free then they should also be compulsory, reducing the problem of non- or symptomatic attendance.

*Make it compulsory for sixty and over to have a regular check-up without having to pay for that, probably it will remove the fear of how much is going to cost. *(13:597)

Reductions in costs were also suggested by those who felt that some payment was necessary. Proposed reductions ranged from twenty to fifty per cent discounts or means tested costs levied according to personal circumstances. However, others considered that care needed to be free in order to remove the very real barrier of cost, regardless of personal circumstances, as was originally the case when the NHS was implemented.

*Actually, it would be helpful for older people to have reduction. If you need treatment you should pay accordingly to your own circumstances. *(66:465; 483)

*If it was free for everybody they would go, because you are on benefit you get it free, because you can't afford to pay but it doesn't mean that with the income she's got coming in, she can afford to go the dentist...it should be totally free after the age of sixty, doesn't matter what your circumstances are. *(36:660)

#### Fear of the cost of dental treatment

Lack of information about the cost of dental treatment led older people to fear the cost of treatment and often to irregular attendance. The example of a participant who realised that they qualified for a flat fee payment in the new system. However, even then there was the need to plan ahead for the payment.

*I couldn't afford to go to the dentist at the cost is now, I would be like I can't go this year, I'll go next year... I know now how much I pay and I pay that much for whatever I've done, so that is quite good really. *(63:486)

### Fear of dental care

Fear of dental care represents the fear of the pain related to dental treatment. This feeling of anxiety associated with going to the dentist was one of the most commonly mentioned issues among dentate older people (Table 2). During the focus groups, participants tried to formulate theories on why people are frightened of going to the dentist. Three factors were deemed important. The first was linked to bad personal experiences of dental treatment, often related to treatments received in childhood. The second factor was related to negative perceptions of dental treatment and encompassed the sound of the drill; unwelcoming or threatening features of the dental surgery itself; anticipation build up caused by long waiting times and negative images portrayed through the media. The final factor identified related to the character of the dentist providing the treatment, and their ability to put the patient at ease. This issue is explored in the final section of the results and was closely linked to fear and its management. The minority of people who did not experience fear of the dentist attributed this to improvements in dental equipment, techniques and pain relief, and the manner of the dentist themselves.

#### Personal bad experiences

For the vast majority of participants, fear of dental treatment was related to their personal experience and often, although not always, originated from childhood experiences.

*A lot of fear really is from when you're young, when I was at school. When you went to a dentist, they never used to use injections. *(23:485)

*I think it's the experience you've had. My son, I took him to the dentist when he was two and he had a very bad experience of taking a tooth out and he still, he's forty something now, he hates going to the dentist and this goes right back to when he was a baby. *(62:198)

While most individuals attributed this fear to bad experience during childhood, bad experiences during adulthood might be just as traumatising. Whilst some people had never conquered their fear, other individuals had overcome it by understanding the introduction of modern equipment and techniques in dentistry.

#### Negative perceptions of dental treatment and the dental environment

Negative perceptions related to dental treatment were considered to enhance fear and anxiety of going to the dentist; these include the sound of the drill:

*The sound of that drill used to drive you mad; I did run out of the dentist once. *(31:154)

unwelcoming or threatening features of the dental surgery itself:

*I don't like when they have got all these photos all around, showing all about teeth, you are waiting to go in and you sit there with these horrible teeth. *(42:155)

anticipation exacerbated by long waiting times:

*I think the fear is in the waiting. When you've got to make an appointment, the next two or three weeks while you are waiting for him to fit you in. *(66:167; 174)

and negative images or references to dentists, portrayed through the media:

*(Talking about a TV programme called 'My family') so watching things like that on the TV can put the fear of going (to the dentist) in people. *(13:520)

Uncertainty was also deemed to be an important factor with fear stemming from the unknown, and fact being replaced by imagined horrors.

### Minimising Barriers Associated with Fear

The main suggestions made for minimising fear of dental treatment focused on moderating the influence of personal bad experiences by acknowledging that treatments have advanced significantly and are less painful than they were in the past; and by reducing negative perceptions of dental treatment through looking at ways of minimising the noise of the drill, reducing waiting times and improving the dental surgery environment.

#### Managing fear

Most participants declared that they had conquered their fear related to previous bad experiences by understanding the improvements in dentistry since their childhood and achieving patterns of attendance rather than avoidance.

*I think as you get older you are not as frightened as when you are a child, are you? The more you go the better is. *(34:835)

*I think dentistry has improved since I was a child during the war. *51:202)

Another option suggested was to provide services differently for older people at day centres and community groups, enabling a wider outreach approach to care. This service would be led by dentists from the dental hospital and should include talking to patients about the benefits of good dental hygiene and regular dental attendance, checking their teeth and taking them to the dental hospital if further treatments were needed:

*Yes, some people have fear, but if they go on a coach with six other people, they won't be so frightened. *(21:463; 467)

Suggestions on improving the environment in which dental care was provided were made relating to the reduction of fear. One participant was particularly scared of the drill and made two suggestions to minimise the sound of dental treatment. The first one referred to 'silent drills', as this individual believed that in other countries the treatment was completely silent.

*In dentistry, in America they do have silent drills, you don't hear the drill at all and why can't we have that sort of things in National Health? *(36:211)

The second suggestion related to the use of earplugs during the treatment to reduce the noise.

*I like what my dentist tried: ear plugs, this is what you pay extra for with private dentists. *(36:716)

The same participants also stated the importance of relaxation techniques in dentistry.

They should know some relaxing system. (36:802)

Furthermore, shorter waiting times for care and between appointments would mean less time for anxiety:

Cutting down the waiting time that means cutting down the fear. (66:181)

There were also suggestions as to how the surgery could be made more welcoming and less intimidating by distractions such as fish tanks, televisions, magazines in a comfortable environment:

I can think of a waiting room comfortable and tidy, sit down and read a magazine. (66:523)

All of these were seen as positive features of the surgery making the experience less frightening and may be summarised as distraction, relaxation, and outreach services.

### Availability of Dental Services

Availability of dental services, or the lack of, was identified as a key barrier to the utilisation of dental services amongst respondents. In the context of the study the concept of availability of dental services refers to the distribution, and the perception of the distribution, of NHS dentists in the three London boroughs of Lambeth, Southwark and Lewisham. There was a widespread perception amongst respondents that there was a shortage of NHS dentists, and that dentists were moving from the public to the private sector in increasing numbers. This perception seemed to be based as much, if not more, on the experiences of 'others' and on the portrayal of dentistry within the media, rather than on personal experience.

Some of the older people interviewed described a personal experience involving the lack of NHS dentists in the area where they lived or concerning NHS dentists moving to the private sector. Both these aspects were related to utilisation through the cost of dental treatment or the fear of the cost of dental treatment. There were several issues associated with the move of NHS dentists to the private sector. Many participants stated they were not able to afford the cost of private dentists and had to look elsewhere for NHS care or stay away.

*I always went to a National Health one, but then they completely went private, I can't afford their prices. *(12:89)

*I should have gone to a National Health (dentist) one but I went to private but it cost me too much money in the end, I'm frightened to go back. *(41:83)

The difficulty of locating another NHS dentist when necessary was also raised:

*We can't go to the dentist because there are not enough NHS dentists. *(21:278)

One individual reported that they changed dentist four times in two or three years because they all went private, but in the end, they found an NHS dentist.

*It was really hard finding a National Health (dentist) but I did! *(63:379)

Perceptions of lack of NHS dentists mainly involved the move towards the private sector as illustrated in the quotes above, and the ability to find a dentist able to take on NHS patients. It was suggested that many dentists in the NHS sector had already reached maximum capacity for NHS patients. So simply finding an NHS dentist was not necessarily sufficient, a fact backed up by the experiences of two respondents.

*I can go to the dentist legless...with no legs, maybe one arm, blind in one eye and they'll say: 'No, I'm sorry, we can't take you on'. *(21:291)

It's very hard and I went up to *** and they said 'no, we're full up and there's nothing we can do about it.' (24:62)

All of the above views were related to informants' personal experience. The generally widespread view of dentists 'turning private' was held by the vast majority of participants, although many did not actually have personal experience of the move.

Every time you find a National Health dentist; they go private, don't they? (65:331)

This was seen as a particular problem for older people who could often not afford private care, and was identified as discrimination.

(They are all going private) which I think it's a bit unfair...(on) elderly people with low incomes. (12:109)

Although a minority view, some participants did acknowledge the availability of NHS dentistry in the area:

*We are in a part of the country where there are quite a few National Health dentists, but I think in other parts of the country people queue like at Starbucks for the dentist. *(51:399)

Even here, however, a problem was identified relating to a lack of knowledge about where the services were located and how to access them.

*... Well, there are some but you've got to find them and know where they are. *(B1:62)

### Minimising Barriers Associated with Availability

Few proposals were suggested to tackle both a lack of NHS dentists but clearly there is a need to address the reported gap in information on how to access those dentists who are practicing NHS dentists and have spaces available (Table 2). One suggestion made was that all dentists should be compelled to take on a certain number or percentage of older people.

*The only way that could be improved is that the government makes all dentists treat pensioners free of charge. They should say that you must have ten pensioners on your books, or twenty pensioners or whatever. *(21:391; 406)

One practical suggestion was that information on available NHS dentists should be displayed at doctors' surgeries.

*I think you should be able to find out through your doctor surgery. GP should be the coordinator of all your health. *(51: 162; 174)

*Your Health Centre should be your centre point for everything. *(B1:413)

Participants from ethnic minority groups also expressed the importance of having information about dental services in different languages available at day centres, which in many cases represented the link between these individuals and society.

### Access to Dental Services

In the context of this study, 'access' refers to all the steps taken by older people in the process of seeking dental care. This area therefore includes all of the different phases from making initial contact with the dental practice to physically being able to get into the surgery and starting treatment. Issues around access emerged as a barrier for older people in seeking and receiving dental care in this study. The issue was not, however, homogenously distributed across the participants and varied significantly according to personal circumstances. Access was a particular issue for people in the 75–84 and 85+ age groups and for people living alone without social support. Problems were identified at all stages from making the initial appointment to travelling to the surgery and accessing the premises. Mobility problems were raised, as was the issue of lack of social support and isolation.

The main problem identified in relation to the appointment system related to the inability to make appointments more than a few weeks in advance. The inability to book the next six-monthly check at the time of the current appointment led to additional stress for people who wanted to plan in advance and not have to worry about forgetting to arrange the next appointment at the right time:

*So they couldn't give me a six months, I've been there for many years, but she said if I phoned up a fortnight before I want to go she'd say if she could get me in. It doesn't make you feel settled, does it? *(31:319; 401)

The uncertainty of not having the next appointment booked was felt to be unsettling. There was also acknowledgement that the appointment system had improved for many people; this was seen as a positive move:

*Dental appointments seem to have improved quite a lot. *(51:385)

Travelling to the dentist may also represent a significant barrier for older people using both private and public transport. In the majority of cases, the older people in this study did not own a car or have access to one; therefore, they relied on public transport or on other people to take them to the dental surgery.

*I want a dentist where I can get to easily because I can't travel too far. *(55:472)

*I can't do walking. *(24:342)

One of the biggest issues that emerged in relation to the appointment was 'timing'. When an appointment was set too early in the morning, older people were not able to use their free bus pass as described in relation to 'costs'. Further costs were also identified for the smaller number of people travelling by car. Again, additional costs such as the London congestion charge and parking costs were identified as pushing the cost of dental treatment out of reach:

*The dentist I go to now is in the congestion charge; you can't go by car, because you can't pay eight pounds every time... when my wife was alive I could take her because she was disabled, I had a disabled disc and everything, so I could park anywhere but now I can't do that, now I have to pay. *(23:309)

The issue of isolation was also raised as a potential problem. This was a significant issue for the oldest participants and raised predominantly by the oldest (85+) males either in relation to travelling to the surgery or in the case of particular treatment such as sedation. In both cases the presence of somebody else was required and the lack of it emphasised their social isolation:

*When you have a sedation somebody has got to go with you, you can't go on your own. *(11:838)

*A lot of elderly people are on their own and the biggest problem is getting somebody to take them to the dentist, nine out of ten of them their children are away. *(21:18)

As the older people in this study were not always able to find someone to accompany them to the dentist, this issue often led to non-attendance. Mobility was another common issue highlighted across the focus groups. A number of the participants had difficulty walking and the lack of disabled access in some surgeries was raised.

*My only thing is that he's up the stairs and it's difficult for me to go up the stairs. *(54:1)

In addition, two carers of older people with significant health and mobility problems were interviewed; both had tried to access domiciliary dental care services on behalf of their clients:

It was a big problem getting somebody (to provide domiciliary care), I spoke to my dentist, I spoke to a lot of local dentists, they were not willing, they said: 'we'll come, we'll come when we've got time' and nothing happened, so then I went to the GP and I said 'look, I'm a bit concerned, you never know' and then the GP phoned up the *** hospital and then they (community dental service) came along. (A1:23)

While this person was able to arrange treatment, a less persistent carer might not have been successful in gaining information on the service. The carer was impressed with the organisation of the service:

It was a perfect service. We did not have to pay at all for that. They wanted to do a perfect job and get the dentures perfect. She came backwards and forwards until the job was done perfect. The convenience of it when you have someone disabled like my mother who cannot move from the waist down and cannot leave the house was marvellous. My mother was delighted with the service and thanked them for coming to the house. (B2:197; 202; 206)

This seems to suggest that, whilst mobility issues are a problem for those who are less disabled, there are services better equipped for those with higher needs, should they persist and find out about the service. Thus, it is the majority, for whom mobility problems are less severe but nevertheless still consider this aspect of access is a barrier to treatment.

### Minimising Barriers Associated with Accessibility

Few suggestions were directly put forward as to how access could be improved; however system changes to address the barriers are outlined in Table 2. Whilst medical visits were already available at some day centres and community groups, there were no equivalent dental services. To solve issues associated with travelling to dental practices and isolation, participants proposed that a mobile dental team should visit day centres and community groups, giving older people a dental examination and taking them to the dental hospital if further treatments were needed. Home visits were also hailed as a good idea for those with more severe mobility problems. This suggests the need for proactive commissioning of care for older people.

*It would be good if we had like a mobile service and then the mobile service can come to the community centre and then we have an interpreter here as well. *(C1:205)

*It's difficult to get to the dentist. What about a dentist coming to you? *(14:808)

For those who were willing and/or able to travel to the surgery, the need for disabled access and overcoming problems with stairs were highlighted:

*It would be nice if it was on the ground floor*. (42:544)

### Characteristics of the Dentist

The final barrier to utilisation of dental services by older people identified in this study related to characteristics of the dentists themselves and their mode of working. Issues raised here related to the communication skills of the dentist and confidence in the dentist as a practitioner; perceptions of the public/private divide in relation to standards of treatment; and experiences of dental hospitals. The personality of the dentist seemed to have a big role in orientating positive or negative feelings towards dental treatment. There were many statements describing how a friendly, polite and professional approach could facilitate positive feelings in older people; on the other hand a hasty manner was seen as a barrier to dental treatment. A range of generally negative views of dentists were displayed:

*(Dentists X) is a bit rough. *(62:233).

*They are not interested in human beings, it's only work coming in, they are only interested in money. *(24:265)

These views were not substantiated or explained even when respondents were questioned further, but appear to be part of a broadly negative view of dentists amongst the informants. The move of NHS dentists into the private sector appeared to add to the negative view of dentists. This was viewed as a sign of their disinterest in people and desire for money:

*They are all going private which is a bit unfair. Elderly people with low incomes...it makes you think they don't want to treat your teeth. *(12:105; 109; 113)

Even those dentists who did remain in the NHS did not escape negative feedback and it was suggested that NHS treatment was a second rate service. Some respondents in this study felt that NHS dentists were not able to provide the same quality of treatment as private ones, issues which they associated with time and care:

*I think they (private dentists) take more time and more care (than NHS dentists). *(36:240)

I find NHS dentists, I mean all the ones I've been to, to be inadequate. If you go privately then they do what I want them to do, I find the National Health dentists do what they want to do. (36:44; 52)

A participant, who was entitled to a reduction on the cost of NHS dental treatments, stated that their dentist was not spending an appropriate amount of time in performing the different treatments needed.

*I think when you're not paying a lot you don't get the same service than people that do pay.*(63:17)

These examples seem to suggest that the dentist has an uphill battle no matter how they choose to practice and suggests that more needs to be done to improve the profile of dentists generally amongst older people and to enhance the importance of caring for older people amongst dentists.

Older people were very aware that the local dental hospitals were training hospitals. Depending on the severity of the case, in dental hospitals, patients may be seen by consultants or supervised students. Many participants stated that they did not feel comfortable with students and they preferred a qualified dentist with more experience:

*Don't forget that dental hospitals are training hospitals. So it's very difficult to get older people to go, they get a bit nervous, they don't wanna see the young student, they wanna see the doctor or the actual dentist rather than a student. *(21:574; 578)

In general, the majority of older people seemed to feel more at ease with a more experienced dentist.

### Minimising Barriers Relating to Characteristics of the Dentist

Recommendations on improvements that dentists themselves could make revolved around the manner of the dentist (demonstrated as professional, interested and caring) and taking time to talk to patients. This can be developed by a range of changes in the system (Table 2). The way in which a dentist welcomes, greets and talks to patients was highlighted as very important to this age group, particularly in helping to reduce fear or anxiety. This includes talking patients' through experiences.

*Some dentists can make you feel totally relaxed straight away not only by being a friendly personality, but incorporating that into a professional approach. *(14:913)

*A nice manner is important to put people at ease, he used to say exactly what he was going to do. *(51:239)

These responses suggest that a professional, polite and friendly manner, in combination with information giving and demonstrating technical skills all help to promote confidence in the patient. Time was also identified as a key factor, both in putting the patient at ease and ensuring the treatment is completed in a satisfactory way.

*I think you've got to have confidence in the dentist because I'm nervous. When I get in there and they are nice to me, I can relax and it's not too bad. *(41:504)

*(It's very important) if they take their time, not rushing as well and take their time with you to see if you are alright. *(34:589)

Views were divided, however, on whether a lack of time was a characteristic of the NHS payment system or of individual dentists. In one case a change in management of patients was blamed:

*He does it all in half an hour and I don't think that's enough...I'd rather go back two or three times. They used to take time, I used to go back to do different things, now this one, he does it all at once and to me that's not good. *(63:26; 242)

Older people welcome a mature professional approach from dentists which combines good communication skills and technically competent clinical care in an unrushed manner, taking on board patient preferences for how care is delivered.

## Discussion

### Limitations of this study

The study population of each of the three boroughs is typical of an inner city socially deprived borough with deprivation scores that fall within the top 60 in the country [25], and exhibit significant ethnic diversity [26]. The informants demonstrates the views of sub-section of older people living in the community who were in the main developing their support through institutions and groups associated with older people or their ethnic group, thus seeking community with peers within the wider community. Although recruitment was continued to achieve geographic, ethnic and where possible age coverage, it was not possible to get many of the oldest population because they are the cohort most likely to be cared for in the community or in care homes. Nonetheless issues pertinent to housebound older people were raised. The authors suggest that its findings are of wider relevance because much of the world's older people live in community settings in a state of economic deprivation [6,8,9], and it is these vulnerable people who most need the support of a state dental care system.

This research took place around the time of NHS dental reforms, which included simplified patient charges. Participants did not express clarity over either system or the changes. The timing in relation to policy changes [27,28], and media exposure to a negative vision of dentistry might have had a crucial role in orienting older people's perceptions, or reinforcing existing perceptions, as many participants thought the reforms would result in a reduction of NHS dental services with no benefit for the citizens. Both these views reflected the negative London and national media coverage at that time in relation to access and availability of NHS care and the suggested need to seek expensive private alternatives.

### Barriers to dental care: summary

This research confirmed that barriers to dental care are widely experienced amongst older people; they are reflected in the low uptake of care locally [14]. Active 'barriers' such as cost, fear, availability, accessibility and features relating to dentists' were reported as well as passive barriers demonstrated by a 'lack of perceived need'. Absence of perceived need, particularly when the individuals have complete dentures, is demonstrated nationally in the findings of the UK adult dental health survey whereby 48% of edentate 65–74 year-olds and 63% of edentate people aged 75 years and over reported that they had not accessed care for over ten years [2]; and despite the fact that past research in this inner city area has revealed a greater unmet need for denture care amongst retired older people when compared with adults in middle age [29]. It is important to note that these views were held despite a healthcare system which provides access to NHS dental care for older people and local availability. Of particular concern is that the barriers identified by Finch et al in the late 1980's remain pertinent to older people in society [17]. However, it could be argued that with the age cohort effect, changing patterns of oral health, and rising costs of care, the issues identified by Finch *et al.*, in the seminal report two decades ago amongst adults in general continued to be very relevant to the current cohort of older people in particular.

Barriers were identified at all stages of the care pathway, from their perception of need, through managing fears about cost and treatment, to identifying available NHS services and gaining access to care at an appropriate time and place. Healthcare in general is free at the point of delivery, as is medication for chronic disease sufferers; however, dental care involves copayments for the majority of older people [30]. In England and Wales the system of patient charges has also changed from 1^st ^April 2006 with some treatment costs going up and some coming down, thus adding to the confusion for older patients. 'Cost' and 'fear of the cost' of dental treatment were mentioned and discussed spontaneously by the majority of participants as discouraging the majority of this age-group from seeking dental treatment until they have a problem. Then it becomes an individual challenge to negotiate what services are available, how, where and when they may be accessed. Although the BDA report 'Older people with teeth' stressed the power of older people in terms of their numbers and wealth [5], this study is a reminder that many socially deprived older people do not consider themselves empowered to access state care.

In a state healthcare system where older people are more likely to use general NHS healthcare, this research contributes to knowledge by outlining why dental care remains less utilised by socially deprived older people and suggests areas for action. This adds to work by Croucher et al. amongst adults in deprived inner city areas [31], and the findings of the English National Working Group which suggest that financial implications are of key significance for this group as many are likely to be living on or below the poverty line, and to have complex treatment needs [7]. Moreover, a quarter of older people live in a situation of relative poverty [8,9]. This research, therefore, supports the literature highlighting the challenge for well developed healthcare systems to 'even out social inequalities in dental health', such as those experienced by older people [6,32]; nationally [33], and globally [6].

### Minimising barriers: summary

In redesigning health services, patient and public views are recognised as important to the effective redesign of healthcare [34,35]. As a result of this research, a series of actions towards minimising barriers has been identified by the public, all of whom have been users of primary dental care at some stage in the past. The findings suggest that 'individual', 'system' and 'societal' action are required to address barriers to dental care, with a particular emphasis on the 'system' (Table 2).

#### Individual action

Individual action includes the persistence required to seek a dentist, from finding the relevant information about NHS care, through to making an appropriate appointment, making the journey for care, agreeing treatment and costs, having the necessary care and coming out with improved oral health and a good patient experience. This might well involve a range of dimensions including the practicalities of making an appointment, travelling, isolation and physical access to premises and managing fears. There is some evidence that fear reduces with age, that the nature of fear also changes [36,37]; and that behavioural and relaxation therapies reduce fear and assist with completion of treatment [38-46]. However, with all this support there needs to be patient motivation to express need, manage fear, attend and complete their care. Individual action also entails being willing, or able, to pay significant costs for NHS dental care. Sometimes these findings suggest that when NHS care was not available, an older person felt obliged to access private dental care which was perceived as 'better' because they were more 'empowered' through paying full costs. However, under the new NHS dental system, patient charges for patients requiring complex work are substantially less [30], which will assist older people.

#### System changes

A wider range of 'system' related changes were identified to facilitate individual experience as presented in Table 2. These involved providing older people with more information about available dental care, dental charges and exemptions.

Addressing the barriers related to 'cost' and 'fear of the cost' of dental treatment was supported by the study participants who went further to suggest that there should be increased NHS resources for older people's dental care in the form of free check-ups or screening in appropriate settings such as day centres. Changes to patient charges for a dental check-up will require national policy to be influenced. It will also be important to monitor the current new GDS system of charges and their impact on older people, both the uptake of care and the nature of care provided. Screening initiatives should be tested as a pilot study for its cost-effectiveness and consider the capacity to deliver subsequent treatment services.

PCTs should assess and commission older peoples dental services in general and domiciliary dental services in particular, based on need [7], and take action to address inequalities in uptake of care. Under new regulations, PCTs have the opportunity to commission care more flexibly within a national framework. Concessions over free checkups have been recommended nationally [5], and are available in Scotland for all patients [47], and more recently in Wales for people over 60 years or over [48]. Such a change requires a national decision and at time of writing there is no evidence that this is likely to happen in England.

Relevant information should be provided for older people and their carers on the availability of NHS dental care, charges, exemptions from co-payments and how to access services, including domiciliary care. This should be widely disseminated to GP practices, pharmacies, carers associations, day centres, libraries and community groups for older people. Primary Care Trusts are required to regularly update the information on dental services on their websites and also on NHS Choices websites. Some Community Dental Services and general dental practices provide a dedicated service for older people. What emerges clearly from the discussions on availability of NHS dentistry is that, whilst the majority of respondents identified a lack of NHS dentists as a particular problem, over half appeared to be basing this perception on the views of others or the media rather than on personal experience. The timing of this study was such that dentistry was experiencing significant negative publicity in London and nationally in parallel with the introduction of the new dental contract.

Although there is much information available on the internet [30], via NHS organisations and other healthcare providers such as GPs, and Age Concern [49], it would appear that further work is required to improve information flows to older people. Community groups are one clear avenue for ongoing information in future. Information on these services should be readily available in formats appropriate to this age cohort. 'Older' older people (over 85 years) in particular usually do not access contemporary networks of information, such as the internet; these people tend to be more passive than younger individuals in seeking out information. Closer links between dentists and health professionals across health and social care can play an important role in improving access and the quality of care.

Characteristics of the dentist in particular were considered important in managing fear of care and enhancing the patient experience. The management of older people's dental care is clearly an issue for both undergraduate and continuing professional development. This should include training for dental health personnel on the needs and the dental life history of older patients and the importance of relating to them in a manner which makes them feel valued and respected. The National Working Group for Older People [7], recommended that 'older people should be entitled to an extended consultation with a dentist to plan out their long-term dental care needs' [7]; clinicians would provide a full assessment of a patient's dental health and the formulation of a comprehensive oral health plan tailored to that individual, including both preventive and clinical care. Furthermore, in light of the ageing population and their oral health needs more dentists should be trained in the dental care of older people and proposals for Special Care Dentistry, which would encompass care for older people by specialists or a dentist with a special interest, will play an important role in the future [7,33]. The physical/mobility needs of this patient group also need to be considered and physical access to dental surgeries must to be ensured along the lines set out by the Disability Discrimination Act [50]; this will particularly be an issue for premises offering Special Care Dentistry in future. Furthermore, there is a clear need to address 'fear', recognising the evidence that behavioural and relaxation therapies reduce fear and assist with completion of treatment [38-46]. Special Care Dentistry will play an important role in providing education and training as well as supporting continuing professional development of the dental team. However, there are funding and policy implications associated with both these initiatives which have yet to be addressed by policy makers. These suggestions, together with action to reduce the barriers identified, are particularly important for the evolving specialty of Special Care Dentistry [33], to address proactively, as they will provide care for vulnerable older people. Access for this group is also an important public health issue [6].

#### Societal change

Finally, at societal level, there is a wider responsibility to support older people in achieving, and where possible retaining, general health and wellbeing, with implications for influencing policy and ensuring that older people have an appropriate support network in society to access healthcare. This study raises the issues of media responsibilities in ensuring balanced reporting, recognising the possible impact of negative reporting on access to healthcare. In particular the relationship between the dental profession and older people clearly to be addressed, with greater mutual understanding so that oral health care can contribute to general health and wellbeing.

## Conclusion

Minimising barriers to dental care in older people involves a range of actions at individual, system and societal level which include: addressing NHS charges; dental team training in the management of older people; when, where and how dental care is provided; information for older people and their carers on available local dental services, dental charges and care pathways.

## Competing interests

The author(s) declare that they have no competing interests.

## Authors' contributions

This study was designed by JG, DW, KJ and SS who obtained NHS project funding and ethics committee approval. Together with EB they formed the research team. EB undertook the fieldwork. EB, SS, DW and JG contributed to this paper. All authors read and approved the final manuscript.

## Pre-publication history

The pre-publication history for this paper can be accessed here:


